# Thyroid hormone sensitivity and diabetes onset: a longitudinal cross-lagged cohort

**DOI:** 10.3389/fendo.2023.1267612

**Published:** 2023-10-16

**Authors:** Cancan Cui, He Sui, Zhijia Wang, Te Zhang, Jia Zheng, Han Yan, Qianyu Li, Zhanhao Mo, Lin Liu

**Affiliations:** China-Japan Union Hospital of Jilin University, Jilin University, Jilin, China

**Keywords:** thyroid hormones sensitivity, type 2 diabetes onset, cross-lagged analysis, TSH (thyroid stimulating hormone), free triiodothyronine (FT3), free thyroxine

## Abstract

**Purpose:**

Thyroid hormones sensitivity is a newly proposed clinical entity closely related with metabolic health. Prior studies have reported the cross-sectional relationship between thyroid hormones sensitivity and diabetes; however, the longitudinal association is unclear to date. We aimed to explore the relationship between impaired thyroid hormone sensitivity at baseline and diabetes onset using a cohort design.

**Methods:**

This study enrolled 7283 euthyroid participants at the first visit between 2008 and 2009, and then annually followed until diabetes onset or 2019. Thyrotropin (TSH), free triiodothyronine (FT3) and free thyroxine (FT4) were measured to calculate thyroid hormone sensitivity by thyroid feedback quantile-based index (TFQI), Chinese-referenced parametric thyroid feedback quantile-based index (PTFQI), thyrotropin index (TSHI), thyrotroph thyroxine resistance index (TT4RI) and FT3/FT4 ratio. Cox proportional hazard model and cross-lagged panel analysis were used.

**Results:**

The mean baseline age was 44.2 ± 11.9 years, including 4170 (57.3%) male. During a median follow-up of 5.2 years, 359 cases developed diabetes. There was no significant association between thyroid hormones sensitivity indices and diabetes onset, and adjusted hazard ratios per unit (95% CIs) were 0.89 (0.65-1.23) for TFQI, 0.91 (0.57-1.45) for PTFQI, 0.95 (0.70-1.29) for TSHI, 0.98 (0.70-1.01) for TT4RI and 2.12 (0.17-5.78) for FT3/FT4 ratio. Cross-lagged analysis supported the temporal association from fasting glucose to impaired thyroid hormones sensitivity indices.

**Conclusions:**

Our findings could not demonstrate that thyroid hormones sensitivity status is a predictor of diabetes onset in the euthyroid population. Elevated fasting glucose (above 7.0 mmol/L) appeared to precede impaired sensitivity indices of thyroid hormones.

## Introduction

By 2021 estimates, diabetes affects 536.6 million people globally, and the prevalence was estimated to rise from 10.5% to 12.2% (783.2 million) in 2045 (1). Approximately, 90-95% of diabetes are type 2 diabetes (2). The global cost of type 2 diabetes and its consequences are large and substantially increasing (3). Thyroid dysfunction and diabetes are closely linked, as the central and peripheral control of thyroid hormones has an impact on glucose homeostasis (4); and insulin sensitivity could modulate the feedback of thyroid hormones in turn (5). Prevalence of thyroid disorders in patients with diabetes is high and vice versa (6). Both hyperthyroidism and hypothyroidism are associated with the development of diabetes (7). From the population studies, evidence suggests that variations of thyroid function even within normal range could be associated with risk of diabetes under complex pathophysiologic mechanisms (8).

Thyroid hormones and thyrotropin (TSH) are inversely correlated under the negative feedback loop of hypothalamic-pituitary-thyroid axis (9). Normal thyroid hormones metabolism and action require adequate cellular receptors (10). The co-occurrence of high thyroid hormones and high TSH represents an acquired resistance to thyroid hormones in the general population (11). Thyroid hormones sensitivity is supposed to tract the metabolic health even in euthyroid population (12, 13). Previous studies have reported the cross-sectional association between thyroid hormones sensitivity and diabetes or prediabetes (12, 14, 15). However, the longitudinal association of thyroid hormones sensitivity with diabetes onset remains unknown to date. Considering the co-existence between thyroid dysfunction and diabetes, the bidirectional relationship needs further interpretation.

Therefore, this study aimed to explore the longitudinal association between thyroid hormones sensitivity indices and incident diabetes using a large cohort.

## Method

### Study population and design

Xiaotangshan Health Examination Cohort is a large-scale dynamic study investigating the risk factors and biomarkers of cardiovascular and metabolic diseases, which was initiated from 2008 at Beijing, China. The recruited participants were required to undertake annual health examinations, face-to-face questionnaire survey and blood sample collection. All plasma samples were tested in a fixed laboratory of Beijing Xiaotangshan Examination Hospital. This current study was a secondary analysis using data from Xiaotangshan Health Examination Cohort. This study included participants between 2008 and 2009 as baseline, and then followed until incident diabetes through repeated examination or censored at the last visit until 2019 following a previous study design (16). Those with pregnant women (n=20) or thyroid dysfunctions (n=1047) were excluded. Finally, a total of 7283 individuals of normal thyroid function were included in the final analysis.

The study was conducted according to the principles of the Declaration of Helsinki and was approved by the Ethics Committee of Xiaotangshan Health Examination Center. All the participants gave an informed consent before participating.

### Data collection and definition

The data of demographic characteristics, lifestyle, diseases histories and medication use were collected *via* a standard questionnaire by our well-trained staff. Educational levels were grouped into illiteracy or primary school (primary), middle school or high school (secondary) and bachelor’s degree or above (third). Active physical activity was defined as having moderate or intense activity at work or during leisure time more than 4 times and 80 minutes weekly. Body mass index (BMI) was calculated as weight in kilograms divided by height in meters squared.

Systolic blood pressure and diastolic blood pressure were presented as the average of two measurements on the right arm using a sphygmomanometer after resting for at least 10 min. Hypertension status was defined as systolic pressure ≥140 mmHg or diastolic pressure ≥90 mmHg, self-reported diagnosis history of hypertension or use of antihypertensive medication (17). Dyslipidemia was defined as triglyceride ≥2.3 mmol/l, total cholesterol ≥6.2 mmol/l, low-density lipoprotein cholesterol ≥4.1 mmol/l, high-density lipoprotein cholesterol <1.0 mmol/l, self-reported diagnosis history of dyslipidemia or use of lipid-lowering medication (18). The estimated glomerular filtration rate (eGFR) was calculated using the Chronic Kidney Disease Epidemiology Collaboration (CKD-EPI 2009) serum creatinine equation (19). Concentration of fasting glucose were tested before breakfast after overnight fasting (no food, except drinking water, for at least 8 hours) by automatic biochemical analyzer Roche Cobas c 701 and SYSMEX HLC-723G8. The onset of diabetes was defined as a composite of fasting glucose ≥7.0 mmol/L or using any glucose-lowering medication or self-reported diagnosis history of diabetes (20).

Serum free triiodothyronine (FT3), free thyroxine (FT4) and thyrotropin (TSH) were measured on the day of sampling using electrochemiluminescence immunoassay (ECLIA) by the auto-analyzer Mindray CL-2000i. Samples were analyzed only when quality control met the acceptable criteria. The inter-assay and intra-assay coefficients of variation (CVs) were all <5.0% for FT3, FT4 and TSH according to the commercial kit instruction (Mindray, Shenzhen, China). The assay-specific reference ranges for FT3, FT4 and TSH were 2.76 to 6.45 pmol/L (to convert FT3 to ng/dL, divided by 15.361), 11.20 to 23.81 pmol/L (to convert FT4 to ng/dL, divided by 12.871) and 0.35 to 5.00 mIU/L. Normal thyroid function is defined as serum FT3, FT4 and TSH levels within the normal range and without use of thyroid hormone supplement.

### Definition of thyroid hormone sensitivity

We calculated thyroid feedback quantile-based index (TFQI) (12) as the cumulative distribution function (cdf) of FT4 and TSH representing the difference between FT4 quantile and the reversed TSH quantile as follows: TFQI = cdf FT4 - (1 - cdf TSH). Parametric TFQI (PTFQI) (21) uses the parameter standard cumulative distribution to improve the applicability to specific population as follows: PTFQI = Φ((FT4 − μFT4)/σFT4) − (1 − Φ((ln TSH − μln TSH)/σln TSH)), where μFT4 = 16.161, σFT4 = 2.131, μln TSH = 0.688, and σln TSH = 0.457 for this current Chinese population. Thyrotropin index (TSHI) (22) was calculated as ln TSH + 0.1345×FT4, and thyrotroph thyroxine resistance index (TT4RI) (23) was calculated as FT4×TSH. Values of TFQI and PTFQI ranges from -1 to 1, with negative and positive values representing good and impaired sensitivity to FT4. For THSI and TT4RI, higher values indicate poor thyroid hormones sensitivity. FT3/FT4 ratio, an index of conversion of thyroid hormones, was calculated as FT3 divided by FT4, and thus lower values indicate less thyroid hormones effect for the same rate of synthesis (24).

### Statistical analysis

Baseline characteristics were described using mean (standard deviation, SD) and frequency (proportion), and the differences were compared by Student’s t-test and Chi-square test according to incident diabetes or not. Distribution of thyroid hormones sensitivity indices were presented using violin plot.

We used Kaplan-Meier curves and Cox proportional hazard model to explore the longitudinal association of baseline thyroid hormones sensitivity indices and incident diabetes. Hazard ratios (HRs) and 95% confidence intervals (CIs) were calculated. Meanwhile, logistics model was used to calculate odds ratios (ORs) for investigating the cross-sectional association between thyroid hormones sensitivity indices and prevalent diabetes. The data of diabetic patients were extracted at the visit time of diagnosis and the data of non-diabetic participants were from the last visit during the follow-up for the cross-sectional analysis. Model 1 was primarily adjusted for age and sex; model 2 was further adjusted for education level, BMI, active physical activity, smoking, hypertension, dyslipidemia, eGFR and fasting glucose. Statistical power of the regression analysis was calculated.

We used the cross-lagged panel to calculate the standard regression coefficients and 95% CIs of baseline thyroid hormones sensitivity indices on subsequent fasting glucose (β1), and the effect of baseline glucose parameters on subsequent thyroid hormones sensitivity indices (β2) after adjusting the auto-regressive effect and covariance. The statistical difference between β1 and β2 was examined using t test. This panel analysis was performed in R package ‘lavaan’. Given the unequal time of follow-up across participants, we performed the analysis adjusting for the follow-up time in model 1. Age, sex and BMI were additionally adjusted in model 2. As a sensitivity analysis, we also included the subgroup (n=1047) of thyroid dysfunctions at baseline and repeated the whole analysis.

The statistical power was calculated using PASS software (version 15). All statistical analyses were performed using R software (version 4.1.0), and a two-sided P value < 0.05 was considered statistically significant.

## Results

### Characteristics

Of 7283 individuals, the mean age was 44.2 (11.9) years, including 4170 (57.3%) males. During a median follow-up of 5.2 years, 359 (4.9%) cases developed diabetes. The detailed baseline characteristics are shown in **Table 1**. We calculated the thyroid hormones sensitivity indices both at baseline and follow-up. **Table S1** shows the distribution of baseline thyroid hormones sensitivity indices stratified by age groups. Of note, there was no significant difference of baseline thyroid hormones sensitivity indices among people with and without incident diabetes during follow-up (**Figure S1**). However, the thyroid hormones sensitivity indices at follow-up were cross-sectionally correlated with prevalent diabetes (**Figure S2**). **Table S2** shows the baseline characteristics after including 1047 participants with thyroid dysfunctions.

**Table 1:** Baseline characteristics of 7283 participants of normal thyroid function.

	Overall	No incident diabetes	Incident diabetes	P value
Participants, No.	7283	6924	359	
Age, years, mean (SD)	44.18 (11.85)	43.72 (11.71)	52.96 (11.15)	<0.001
Median (min-max)	44 (20-85)	44 (20-85)	53 (24-85)	<0.001
Sex, Male, n (%)	4170 (57.3)	3908 (56.4)	262 (73.0)	<0.001
Educational level, n (%)				0.016
Primary	586 (8.0)	543 (7.8)	43 (12.0)	
Secondary	4594 (63.1)	4372 (63.1)	222 (61.8)	
Third	2103 (28.9)	2009 (29.0)	94 (26.2)	
Active physical activity a, n (%)	2146 (29.5)	2038 (29.4)	108 (30.1)	0.838
Current smoking, n (%)	1663 (22.8)	1579 (22.8)	84 (23.4)	0.844
BMI b, kg/m2	25.00 (3.49)	24.90 (3.47)	26.90 (3.36)	<0.001
Hypertension, n (%)	1182 (16.2)	1081 (15.6)	101 (28.1)	<0.001
Dyslipidemia, n (%)	2271 (31.2)	2097 (30.3)	174 (48.5)	<0.001
Thyroid nodule, n (%)	2245 (30.8)	2093 (30.2)	152 (42.3)	<0.001
Family history of diabetes, n (%)	493 (6.8)	463 (6.7)	30 (8.4)	0.263
Fasting glucose, mmol/L	5.19 (0.53)	5.15 (0.48)	6.08 (0.64)	<0.001
eGFR, mL/min per 1.73 m^2^	95.83 (18.49)	95.92 (18.44)	94.12 (19.31)	0.071
TSH, mIU/L	2.20 (0.95)	2.20 (0.95)	2.19 (0.96)	0.963
FT3, pmol/L	4.93 (0.60)	4.93 (0.60)	4.94 (0.62)	0.598
FT4, pmol/L	16.16 (2.13)	16.16 (2.13)	16.10 (2.15)	0.573

Data are presented as mean (SD) or number (%), as appropriate.

P values were calculated using Chi-square test for categorical variables and Student’s t-test for continuous variables.

SD, standard deviation; BMI, body mass index; eGFR, estimated glomerular filtration rate; TSH, thyrotropin; FT3, free triiodothyronine; FT4, free thyroxine.

SI conversion factors: To convert fasting plasma glucose to mg/dL, multiply by 18.0; to convert FT3 to ng/dL, divided by 15.361; to convert FT4 to ng/dL, divided by 12.871.

a Active physical activity was defined as having moderate or intense activity at work or during leisure time more than 4 times and 80 minutes weekly.

b Calculated as weight in kilograms divided by height in meters squared (missing data in 679 participants).

Normal thyroid function is defined as serum TSH, FT3 and FT4 levels within the normal range.

### Cross-sectional and longitudinal association of thyroid hormones sensitivity indices with diabetes onset

**Figure 1:**
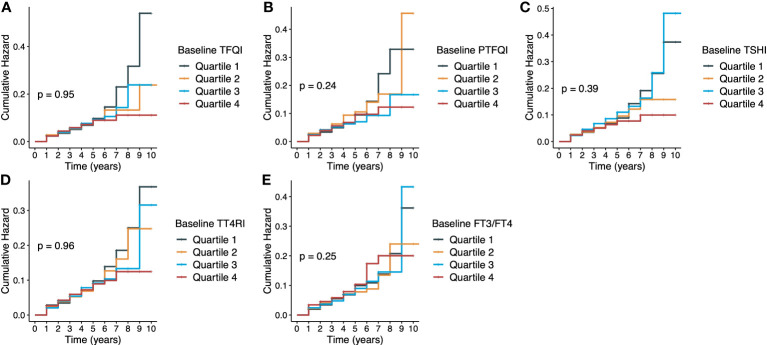
Kaplan-Meier curves of diabetes according to thyroid hormones sensitivity indices. **(A)** TFQI and diabetes; **(B)** PTFQI and diabetes; **(C)** TSHI and diabetes; **(D)** TT4RI and diabetes; **(E)** FT3/FT4 ratio and diabetes. TFQI, thyroid feedback quantile-based index; PTFQI, Chinese-referenced parametric thyroid feedback quantile-based index; TSHI, thyrotropin index; TT4RI, thyrotroph thyroxine resistance index; FT3, free triiodothyronine; FT4, free thyroxine.

**Figure 1** presents the Kaplan-Meier curves of diabetes onset according to the quartile groups of baseline thyroid hormones sensitivity indices. There were no significant longitudinal associations between five thyroid hormones sensitivity indices and incident diabetes, and HRs (95% CIs) of per-unit were 0.89 (0.65-1.23) for TFQI, 0.91 (0.57-1.45) for PTFQI, 0.95 (0.70-1.29) for TSHI, 0.98 (0.70-1.01) for TT4RI and 2.12 (0.17-5.78) for FT3/FT4 ratio in the fully adjusted model (**Table 2**). Age, BMI, eGFR and fasting glucose were positively associated with diabetes onset, while people with higher education level had lower risk of incident diabetes (**Table S3**). No statistical significance was demonstrated in any strata in the subgroup analysis of age and sex (**Figure S3**). Following a previous study (12), it was assumed that people on the highest quartile of TFQI had 73.0% higher risk of diabetes. Given a 5.0% incidence rate of diabetes in our population, the current sample size of 7283 could achieve a statistical power of 0.992. Of note, there were significant cross-sectional associations between thyroid hormones sensitivity indices with prevalent diabetes at the end of follow-up as shown in **Table S4**. Results were consistent when repeating the analysis after including 1047 participants with thyroid dysfunctions at baseline **Table S5**.

**Table 2:** Association of baseline thyroid hormones sensitivity indices with incident diabetes.

	Model 1			Model 2		
	HR	95% CI	P	HR	95% CI	P
TFQI (continuous)	0.828	0.616-1.113	0.210	0.894	0.650-1.229	0.489
Quartile 1 (-0.928 to -0.224)	Ref					
Quartile 2 (-0.225 to 0.016)	0.968	0.724-1.293	0.823	0.859	0.634-1.164	0.326
Quartile 3 (0.017 to 0.261)	0.915	0.682-1.228	0.556	0.918	0.677-1.244	0.579
Quartile 4 (0.262 to 0.925)	0.904	0.674-1.212	0.500	0.918	0.675-1.248	0.584
PTFQI (continuous)	0.709	0.458-1.096	0.122	0.907	0.568-1.447	0.681
Quartile 1 (-0.678 to -0.193)	Ref					
Quartile 2 (-0.194 to -0.014)	1.146	0.864-1.521	0.345	1.079	0.802-1.452	0.616
Quartile 3 (-0.015 to 0.163)	0.820	0.605-1.110	0.199	0.790	0.571-1.092	0.154
Quartile 4 (0.164 to 0.756)	0.898	0.665-1.214	0.485	1.066	0.780-1.457	0.687
TSHI (continuous)	0.918	0.750-1.125	0.410	0.951	0.770-1.175	0.643
Quartile 1 (0.926 to 2.539)	Ref					
Quartile 2 (2.540 to 2.887)	1.028	0.766-1.380	0.853	0.952	0.702-1.292	0.753
Quartile 3 (2.888 to 3.216)	1.099	0.826-1.462	0.519	0.974	0.724-1.310	0.861
Quartile 4 (3.217 to 4.576)	0.879	0.650-1.188	0.401	0.825	0.603-1.128	0.228
TT4RI (continuous)	0.998	0.992-1.005	0.651	0.998	0.770-1.005	0.526
Quartile 1 (5.772 to 23.706)	Ref					
Quartile 2 (23.707 to 32.662)	1.031	0.769-1.382	0.837	0.983	0.702-1.330	0.910
Quartile 3 (32.663 to 44.258)	0.948	0.707-1.271	0.720	0.910	0.724-1.238	0.549
Quartile 4 (44.259 to 106.190)	0.987	0.739-1.319	0.932	0.876	0.603-1.186	0.390
FT3/FT4 (continuous)	1.780	0.436-2.094	0.069	2.121	0.174-5.780	0.555
Quartile 1 (0.148 to 0.278)	Ref					
Quartile 2 (0.279 to 0.304)	1.115	0.826-1.505	0.477	0.837	0.612-1.145	0.266
Quartile 3 (0.305 to 0.333)	1.147	0.849-1.551	0.371	0.902	0.655-1.242	0.528
Quartile 4 (0.334 to 0.537)	1.436	1.072-1.925	0.015	1.065	0.782-1.450	0.691

HR, hazard ratio; CI, confidence interval; TFQI, thyroid feedback quantile-based index; PTFQI, Chinese-referenced parametric thyroid feedback quantile-based index; TSHI, thyrotropin index; TT4RI, thyrotroph thyroxine resistance index; FT3, free triiodothyronine; FT4, free thyroxine; BMI, body mass index; eGFR, estimated glomerular filtration rate.

Model 1: age and sex adjusted; model 2: age, sex, education level, BMI, physical activity, smoking, hypertension, hyperlipidemia, eGFR and fasting glucose adjusted.

### Cross-lagged panel analyses

**Figure 2:**
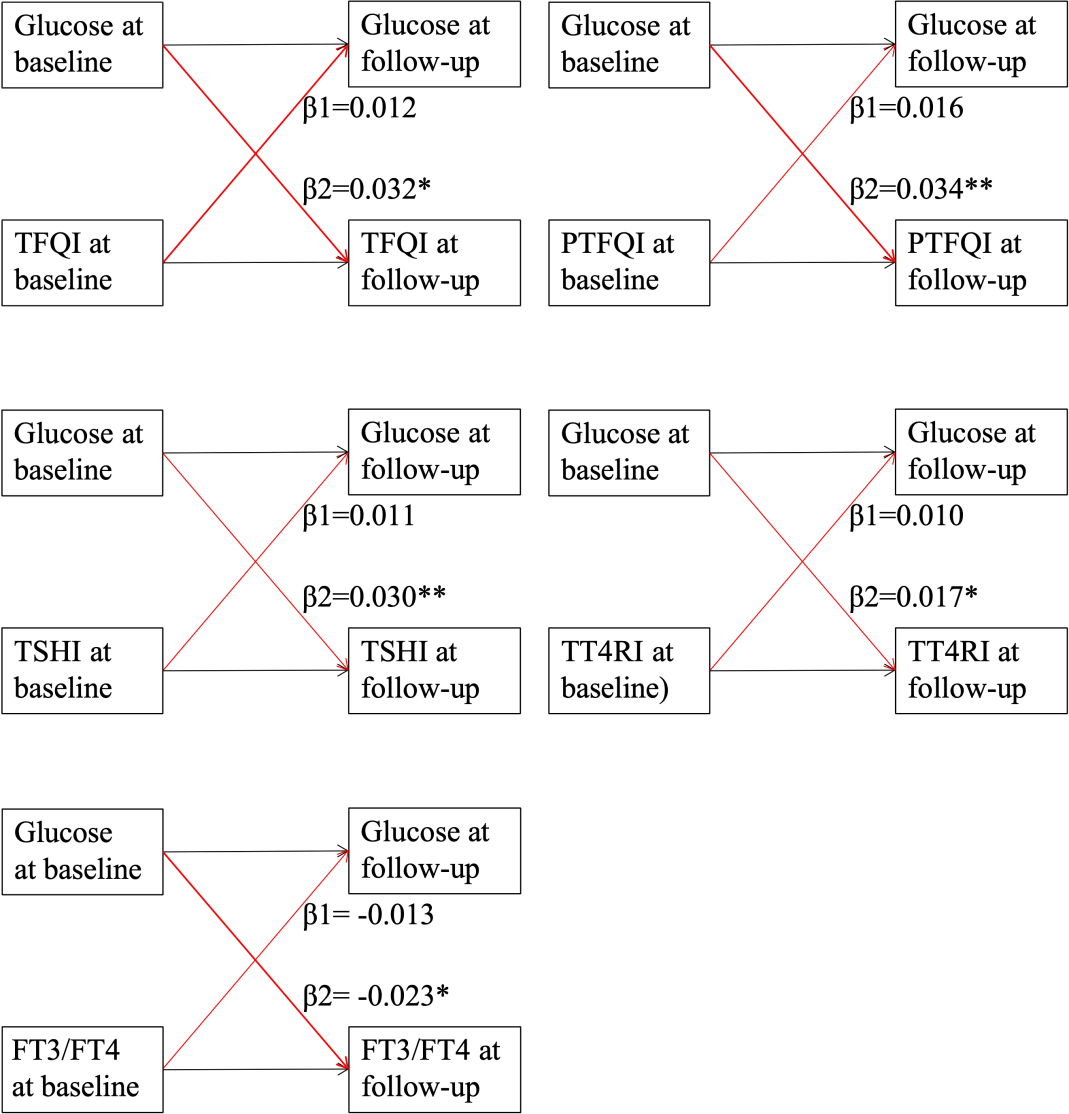
Cross-lagged panel analysis of thyroid hormones sensitivity indices with fasting glucose. Continuous levels of thyroid hormones sensitivity indices and fasting glucose at two time points (baseline and last follow-up) were used in the cross-lagged panel. TFQI, thyroid feedback quantile-based index; PTFQI, Chinese-referenced parametric thyroid feedback quantile-based index; TSHI, thyrotropin index; TT4RI, thyrotroph thyroxine resistance index; FT3, free triiodothyronine; FT4, free thyroxine. β1: thyroid hormones sensitivity index at baseline→ fasting glucose at follow-up; β2: fasting glucose at baseline→thyroid hormones sensitivity index at follow-up. ** indicates P value <0.01; * indicates P value <0.05.

**Figure 2** presents the cross-lagged panel analysis between thyroid hormones sensitivity indices and fasting glucose. The adjusted standardized correlation coefficients (95% CI) of baseline thyroid hormones sensitivity indices to follow-up fasting glucose (β1) was 0.012 (-0.007, 0.031) for TFQI, 0.016 (-0.003, 0.036) for PTFQI, 0.011 (-0.007, 0.032) for TSHI, 0.010 (-0.005, 0.029) for TT4RI and -0.013 (-0.027, 0.008) for FT3/FT4 ratio. The effects of baseline fasting glucose to subsequent thyroid hormones sensitivity indices (β2) were 0.032 (0.012-0.057) for TFQI, 0.034 (0.011, 0.056) for PTFQI, 0.030 (0.004, 0.050) for TSHI, 0.017 (0.011, 0.023) for TT4RI and -0.023 (-0.035, -0.011) for FT3/FT4 ratio (**Table 3**). After excluding those using anti-diabetic medication at the follow-up survey, the results remained almost consistent except that the adjusted standardized correlation coefficients between TT4RI and fasting glucose became insignificant, although the unadjusted coefficient from fasting glucose to TT4RI was still significant (**Table S6**).

**Table 3:** Cross-lagged standard regression coefficient of thyroid hormones sensitivity indices with fasting glucose.

	Model 1				Model 2			
	β	P value	95% CI		β	P value	95% CI	
			lower	upper			lower	upper
TFQI and Glucose *								
Glucose.x→TFQI.y	0.044	<0.001	0.020	0.063	0.032	0.014	0.012	0.057
TFQI.x→Glucose.y	0.014	0.085	0.001	0.032	0.012	0.162	-0.007	0.031
PTFQI and Glucose *								
Glucose.x→PTFQI.y	0.054	<0.001	0.034	0.073	0.034	0.001	0.011	0.056
PTFQI.x→Glucose.y	0.018	0.027	0.001	0.037	0.016	0.069	-0.003	0.036
TSHI and Glucose *								
Glucose.x→TSHI.y	0.043	<0.001	0.021	0.065	0.030	0.008	0.004	0.050
TSHI.x→Glucose.y	0.012	0.195	-0.009	0.031	0.011	0.249	-0.007	0.032
TT4RI and Glucose *								
Glucose.x→TT4RI.y	0.027	0.032	0.001	0.055	0.017	0.023	0.011	0.023
TT4RI.x→Glucose.y	0.008	0.320	-0.006	0.028	0.010	0.249	-0.005	0.029
FT3/FT4 and Glucose *								
Glucose.x→FT3/FT4.y	-0.036	0.001	-0.054	-0.016	-0.023	0.041	-0.035	-0.011
FT3/FT4.x→Glucose.y	-0.011	0.285	-0.027	0.010	-0.013	0.140	-0.027	0.008

CI, confidence interval; TFQI, thyroid feedback quantile-based index; PTFQI, Chinese-referenced parametric thyroid feedback quantile-based index; TSHI, thyrotropin index; TT4RI, thyrotroph thyroxine resistance index; FT3, free triiodothyronine; FT4, free thyroxine.

.x indicates measurements at baseline and.y indicates measurements at last time follow-up.

*indicates significant difference (P <0.001) in the cross-lagged coefficients using t test comparing the coefficients from thyroid sensitivity index to glucose and from glucose to thyroid sensitivity index.

Model 1: follow-up time adjusted; model 2: adjusted for age, sex, BMI and follow-up time.

## Discussion

In this study, we explored the longitudinal relationship between thyroid hormones sensitivity indices and diabetes onset using a large cohort. Our findings could not demonstrate that thyroid hormones sensitivity status is a predictor of diabetes onset. The cross-lagged panel analysis indicated that elevated fasting glucose level (above 7.0 mmol/L) precedes impaired sensitivity indices of thyroid hormones. The results remained consistent among multiple subgroups and sensitivity analyses.

The cross-sectional association between reduced sensitivity to thyroid hormones and metabolic abnormalities have been reported in previous studies. Laclaustra et al. proposed the TFQI index to indicate the resistance to thyroid hormones and found that TFQI index was associated with prevalent diabetes and metabolic syndrome (12). Mehran et al. reported that TFQI and PTFQI were related with high blood pressure and known diabetes among 5124 euthyroid subjects (14). Liu et al. found that decreased sensitivity to thyroid hormones is associated with lower risk of prediabetes (15). Yu et al. reported the significant association between impaired sensitivity to thyroid hormones and elevated blood glucose (above 7.0 mmol/L) among people with coronary heart diseases (25). Our study confirmed the cross-sectional association between thyroid hormones sensitivity and prevalent diabetes. However, the longitudinal and bidirectional association of thyroid hormones sensitivity with diabetes remains unknown to date. Using a cohort design, our findings could not demonstrate that thyroid hormones sensitivity indices are independently associated with the risk of diabetes onset. The insignificant relationship could also partially attribute to the fact that people developed diabetes trend to have more risk factors, such as being older, having higher BMI and lipids levels, having been diagnosed with hypertension (seen in **Table 1**). These coexisting factors could probably attenuate with effect of thyroid hormones sensitivity on diabetes. The opposite results between our cross-sectional and longitudinal analyses suggested reverse causation between thyroid hormones sensitivity and diabetes, which was supported by our cross-lagged panel analysis. Moreover, Mehran et al. also assumed that the interpretation for diabetes should be concerned with cautions, as there was no significant cross-sectional relationship between TFQI and new-onset diabetes (14). Of note, it has been recognized that TSH upper limit increases with normal ageing and the elderly may be adapted to the physiological increase (26). Our previous study also showed that the elderly seem not sensitive to high TSH regarding metabolic syndrome (27). In the subgroup analysis of age, we observed consistent results that thyroid hormones sensitivity indices are not independent risk factors of diabetes onset. Similarly, the cross-sectional associations of thyroid hormones sensitivity indices with other metabolic traits have been reported. We hypothesized that metabolic disorders including type 2 diabetes could lead to acquired resistance to thyroid hormones. The phenomenon of co-existing metabolic disorders and impaired thyroid hormones sensitivity warrants further research, especially for the bidirectional relationship between metabolic health and thyroid hormones sensitivity.

In the cross-lagged panel analysis, the findings supported that fasting glucose appeared to precede the impaired thyroid hormones sensitivity, indicating elevated fasting glucose (above 7.0 mmol/L) may drive acquired resistance of thyroid hormones. Previous studies pointed that the prevalence of hyperthyroidism and hypothyroidism in patients with diabetes is higher than in the general population (28, 29). Diabetes could also aggravate the progression from subclinical to overt hypothyroidism (30). A review emphasized the urgent need to screen the onset of thyroid diseases in patients with diabetes (4). Our study suggested that diabetes potentially leads to the resistance of thyroid hormones in an euthyroid state. Consistently, studies have reported that the longer duration of diabetes is an important risk factor for the development of thyroid dysfunction (31, 32). The fact should be acknowledged is that the thyroid hormones sensitivity is defined by the mathematical interpretations of thyroid hormones, but not experimentally validated measurements. Both high FT4 and high TSH are present in the resistance to thyroid hormones. A mild acquired resistance to thyroid hormones might occur in the euthyroid population, of which the clinical significance and physiological importance still need more evidence to be addressed. All these resistance to thyroid hormone indices measure central sensitivity or resistance, i.e., the grade of pituitary gland inhibition by FT4 levels (12). Although the thyroid hormones sensitivity index may not be an independent risk predictor of diabetes, it could be a consequence of metabolic disorders reversely as observed in our study. Our results offer an explanation for thyroid profiles commonly found in patients with diabetes, that is at the population level, diabetes is associated with the subsequent resistance to thyroid hormones. It suggests that there might be other underlying mechanisms linking diabetes and resistance to thyroid hormone, which is also indicated in previous studies (33, 34). People of diabetes or other metabolic disorders should be aware of the thyroid function resistance and its consequences, including lipid pattern (35), renal and liver functions (13, 36), atherosclerosis (37), cognition performance (38) and mortality (12). Meanwhile, previous studies have demonstrated a close association between thyroid hormones sensitivity and metabolic disorders based on cross-sectional designs (39). The causal and temporal relationship needs more data to interpret the clinical relevance of thyroid hormones sensitivity in depth.

There are several potential mechanisms that could explain the relationship between glucose metabolism and thyroid hormones resistance. It has been indicated that only overt thyroid dysfunction leads to the disruption of glucose metabolism *via* insulin resistance (40, 41), which partially explains that thyroid hormones resistance under an euthyroid state could not predict diabetes onset. Conversely, our study indicated that hyperglycemia precedes thyroid hormones resistance, as hyperglycemia can control the conversion from thyroxine to triiodothyroxine in peripheral tissues and interfere the thyroid hormones (42, 43). In addition, inflammation may be involved in the pathogenesis of diabetes and thyroid hormones homeostasis. Studies have shown that people with abnormal glucose metabolism have high levels of inflammatory cytokines (44). The crosstalk between the cytokine IL-37 and thyroid hormones could be the key regulatory mechanism that justifies the glucose metabolic effects (45). The exact pathophysiological mechanism of glucose metabolism on thyroid hormones resistance needs further research.

The results should be interpreted in the context of limitations. First, this is an observational study design, and we were unable to claim the causal association between thyroid hormones sensitivity and diabetes. The observed results require further validation in other populations. Whether controlling glucose level could improve the thyroid hormones sensitivity status needs further clinical research. As an observational study, there are potential confounding factors that are not considered in this study, such as effect of specific medication use on TSH synthesis or secretion (glucocorticoid treatment, anticonvulsants and antidepressant medications). Second, this study is a large population study based on a health examination cohort, and the data of 2-hour postprandial blood glucose (2hPBG) and HbA1c were not available. The incidence of diabetes could be underestimated as 2hPBG and HbA1c can diagnose more people with diabetes compared with fasting glucose (1, 46), probably causing estimation deviation of the effect size. Further studies are needed to clarify the association between glucose dysregulation and thyroid hormones sensitivity in depth. Third, the follow-up period is relatively short, which is probably not enough to observe the effect of impaired thyroid hormones sensitivity on the risk of diabetes.

In summary, our findings could not demonstrate that thyroid hormones sensitivity status is a predictor of diabetes onset, suggesting diabetes may precede impaired sensitivity indices of thyroid hormones, which needs validation in further studies.

## Data availability statement

The raw data supporting the conclusions of this article will be made available by the authors, without undue reservation.

## Ethics statement

The studies involving humans were approved by Committee of Xiaotangshan Health Examination Center. All the participants gave an informed consent before participating. The studies were conducted in accordance with the local legislation and institutional requirements. Written informed consent for participation was not required from the participants or the participants’ legal guardians/next of kin in accordance with the national legislation and institutional requirements.

## Author contributions

CC: Formal Analysis, Writing – original draft. HS: Writing – original draft. ZW: Methodology, Writing – review & editing. TZ: Writing – review & editing, Validation. JZ: Writing – review & editing, Resources. HY: Writing – review & editing, Formal Analysis. QL: Writing – review & editing, Data curation. ZM: Writing – review & editing, Supervision. LL: Supervision, Writing – review & editing.
