# Supplementary material for: Thyroid hormone sensitivity and diabetes onset: a longitudinal cross-lagged cohort
Source: Front Endocrinol (Lausanne). 2023 Oct 16;14:1267612. doi: 10.3389/fendo.2023.1267612 (PMC10613705; doi:10.3389/fendo.2023.1267612)
Supplement: Supplementary file 1 [file DataSheet_1.docx]

**Supplemental Material**

**Thyroid hormone sensitivity and diabetes onset: a longitudinal cross-lagged cohort**

**1 Supplemental Tables**

**Table S1:** Distribution of thyroid hormone sensitivity indices at baseline stratified by age groups.

**Table S2:** Baseline characteristics of 8330 participants.

**Table S3:** Full regression results for association of thyroid hormone sensitivity indices with incident diabetes.

**Table S4:** Cross-sectional association between thyroid hormone sensitivity indices and prevalent diabetes at the last time follow-up.

**Table S5:** Association of baseline thyroid hormone sensitivity indices with incident diabetes.

**Table S6**: Cross-lagged standard regression coefficient of thyroid hormone sensitivity indices and fasting glucose after excluding 63 participants using anti-diabetic mediation.

**2 Supplemental Figures**

**Figure S1:** Distribution of baseline thyroid hormone sensitivity indices according incident diabetes or not.

**Figure S2:** Distribution of thyroid hormone sensitivity indices according prevalent diabetes or not at the last time point of follow-up.

**Figure S3:** Association of baseline thyroid hormone sensitivity indices with incident diabetes among subgroups of age and sex.

**1 Supplemental Tables**

**Table S1**: Distribution of thyroid hormone sensitivity indices at baseline stratified by age groups.

|  | Age, years | | |
| --- | --- | --- | --- |
|  | < 40 | 40-59 | ≥ 60 |
| Participants, No. | 2550 | 4132 | 601 |
| TFQI | 0.03 [-0.20, 0.30] | 0.01 [-0.23, 0.25] | 0.00 [-0.23, 0.24] |
| PTFQI | 0.00 [-0.19, 0.18] | -0.02 [-0.20, 0.15] | -0.02 [-0.20, 0.14] |
| TSHI | 2.91 [2.57, 3.24] | 2.87 [2.52, 3.20] | 2.90 [2.54, 3.21] |
| TT4RI | 33.19 [24.33, 44.55] | 32.17 [23.44, 43.85] | 33.36 [23.28, 45.51] |
| FT3/FT4 | 0.31 [0.28, 0.34] | 0.30 [0.28, 0.33] | 0.30 [0.27, 0.33] |

Data are presented as median [IQR].

Abbreviation: IQR, interquartile range; TFQI, thyroid feedback quantile-based index; PTFQI, Chinese-referenced parametric thyroid feedback quantile-based index; TSHI, thyrotropin index; TT4RI, thyrotroph thyroxine resistance index; FT3, free triiodothyronine; FT4, free thyroxine.

**Table S2:** Baseline characteristics of 8330 participants.

|  | Overall | No incident diabetes | Incident diabetes | P value |
| --- | --- | --- | --- | --- |
| Participants, No. | 8330 | 7907 | 423 |  |
| Age, years | 44.20(11.98) | 43.72(11.83) | 53.07(11.15) | <0.001 |
| Sex, Male, n (%) | 4656(55.9) | 4352(55.0) | 304(71.9) | <0.001 |
| Educational level, n (%) |  |  |  |  |
| Primary | 705(8.5) | 650(8.2) | 55(13.0) | 0.002 |
| Secondary | 5231(62.8) | 4970(62.9) | 261(61.7) |  |
| Third | 2394(28.7) | 2287(28.9) | 107(25.3) |  |
| Physical activity, n (%) | 2444(29.3) | 2314(29.3) | 130(30.7) | 0.554 |
| Current smoking, n (%) | 1868(22.4) | 1764(22.3) | 104(24.6) | 0.301 |
| BMI ^a^, kg/m^2^ | 25.00(3.52) | 24.89(3.49) | 27.01(3.43) | <0.001 |
| Self-reported hypertension, n (%) | 543(6.5) | 497(6.3) | 46(10.9) | <0.001 |
| Self-reported dyslipidemia, n (%) | 226(2.7) | 203(2.6) | 23(5.4) | 0.001 |
| Fasting glucose, mmol/L | 5.19(0.53) | 5.14(0.48) | 6.08(0.62) | <0.001 |
| eGFR, mL/min per 1.73 m^2^ | 95.58(18.40) | 95.71(18.36) | 93.30(18.86) | 0.009 |

Data are presented as mean (SD) or number (%), as appropriate.

P values were calculated using Chi-square test for categorical variables and Student's t-test for continuous variables.

Abbreviation: SD, standard deviation; BMI, body mass index; eGFR, estimated glomerular filtration rate; TSH, thyrotropin; FT4, free thyroxine.

SI conversion factors: To convert fasting plasma glucose to mg/dL, multiply by 18.0; to convert free FT4 to ng/dL, divided by 12.871.

^a^ Calculated as weight in kilograms divided by height in meters squared (missing data in 759 participants).

**Table S3:** Significant terms in full regression for association between thyroid hormone sensitivity indices and incident diabetes.

|  | HR ^a^ | P value | 95% CI | |
| --- | --- | --- | --- | --- |
|  |  |  | lower | upper |
| TFQI | 0.894 | 0.489 | 0.650 | 1.229 |
| Age | 1.034 | <0.001 | 1.023 | 1.044 |
| BMI | 1.040 | 0.032 | 1.003 | 1.077 |
| eGFR | 1.008 | 0.005 | 1.003 | 1.014 |
| Fasting glucose | 10.769 | <0.001 | 8.866 | 13.081 |
| Education Third (Ref: Primary) | 0.658 | 0.034 | 0.447 | 0.968 |
| PTFQI | 0.907 | 0.681 | 0.568 | 1.447 |
| Age | 1.034 | <0.001 | 1.023 | 1.044 |
| BMI | 1.039 | 0.034 | 1.003 | 1.077 |
| eGFR | 1.009 | 0.005 | 1.003 | 1.014 |
| Fasting glucose | 10.770 | <0.001 | 8.866 | 13.082 |
| Education Third (Ref: Primary) | 0.655 | 0.033 | 0.445 | 0.966 |
| TSHI | 0.951 | 0.643 | 0.770 | 1.175 |
| Age | 1.034 | <0.001 | 1.023 | 1.044 |
| BMI | 1.040 | 0.032 | 1.003 | 1.077 |
| eGFR | 1.009 | 0.005 | 1.003 | 1.014 |
| Fasting glucose | 10.772 | <0.001 | 8.868 | 13.085 |
| Education Third (Ref: Primary) | 0.653 | 0.031 | 0.444 | 0.961 |
| TT4RI | 0.998 | 0.526 | 0.770 | 1.005 |
| Age | 1.034 | <0.001 | 1.023 | 1.044 |
| BMI | 1.040 | 0.030 | 1.003 | 1.078 |
| eGFR | 1.009 | 0.005 | 1.003 | 1.014 |
| Fasting glucose | 10.783 | <0.001 | 8.868 | 13.098 |
| Education Third (Ref: Primary) | 0.653 | 0.030 | 0.444 | 0.961 |
| FT3/FT4 | 2.121 | 0.555 | 0.174 | 25.780 |
| Age | 1.034 | <0.001 | 1.024 | 1.045 |
| BMI | 1.038 | 0.038 | 1.002 | 1.076 |
| eGFR | 1.009 | 0.004 | 1.003 | 1.015 |
| Fasting glucose | 10.753 | <0.001 | 8.852 | 13.061 |
| Education Third (Ref: Primary) | 0.657 | 0.033 | 0.446 | 0.968 |

Abbreviation: HR, hazard ratio; CI, confidence interval; TFQI, thyroid feedback quantile-based index; PTFQI, Chinese-referenced parametric thyroid feedback quantile-based index; TSHI, thyrotropin index; TT4RI, thyrotroph thyroxine resistance index; FT3, free triiodothyronine; FT4, free thyroxine; BMI, body mass index; eGFR, estimated glomerular filtration rate.

^a^ Age, sex, education level, BMI, physical activity, smoking, hypertension, hyperlipidemia, eGFR and fasting glucose were adjusted in regression analysis.

**Table S4**: Cross-sectional association between thyroid hormone sensitivity indices and prevalent diabetes at the last time follow-up.

|  | Model 1 | | | Model 2 | | |
| --- | --- | --- | --- | --- | --- | --- |
|  | OR ^a^ | 95% CI | P value | OR ^a^ | 95% CI | P value |
| TFQI | 2.090 | 1.623-2.691 | <0.001 | 1.976 | 1.497-2.608 | <0.001 |
| PTFQI | 2.673 | 1.957-3.651 | <0.001 | 2.399 | 1.693-3.398 | <0.001 |
| TSHI | 1.394 | 1.219-1.593 | <0.001 | 1.388 | 1.190-1.619 | <0.001 |
| TT4RI | 1.005 | 1.002-1.009 | 0.002 | 1.007 | 1.190-1.011 | <0.001 |
| FT3/FT4 | 0.097 | 0.030-0.314 | <0.001 | 0.172 | 0.050-0.596 | 0.006 |

Abbreviation: OR, odds ratio; CI, confidence interval; TFQI, thyroid feedback quantile-based index; PTFQI, Chinese-referenced parametric thyroid feedback quantile-based index; TSHI, thyrotropin index; FT3, free triiodothyronine; FT4, free thyroxine; TT4RI, thyrotroph thyroxine resistance index; BMI, body mass index; eGFR, estimated glomerular filtration rate.

All variables used were those at the end point of follow-up; logistics model was used to calculated the OR and 95% CI.

Model 1: age and sex adjusted; model 2: age, sex, education level, BMI, physical activity, smoking, hypertension, dyslipidemia, eGFR and fasting glucose adjusted.

^a^ OR was calculated for per unit of each thyroid hormone sensitivity index.

**Table S5:** Association of baseline thyroid hormone sensitivity indices with incident diabetes.

|  | Model 1 | | | Model 2 | | |
| --- | --- | --- | --- | --- | --- | --- |
|  | HR | 95% CI | P value | HR | 95% CI | P value |
| TFQI (continuous) | 0.82 | 0.632-1.064 | 0.135 | 0.914 | 0.691-1.21 | 0.531 |
| Quartile 1 | Ref |  |  |  |  |  |
| Quartile 2 | 0.872 | 0.663-1.147 | 0.327 | 0.759 | 0.57-1.01 | 0.059 |
| Quartile 3 | 0.908 | 0.694-1.187 | 0.479 | 0.911 | 0.691-1.201 | 0.509 |
| Quartile 4 | 0.856 | 0.653-1.123 | 0.262 | 0.896 | 0.675-1.189 | 0.447 |
| PTFQI (continuous) | 0.718 | 0.497-1.037 | 0.077 | 0.918 | 0.618-1.365 | 0.672 |
| Quartile 1 | Ref |  |  |  |  |  |
| Quartile 2 | 1.05 | 0.805-1.37 | 0.719 | 0.988 | 0.748-1.303 | 0.929 |
| Quartile 3 | 0.826 | 0.626-1.09 | 0.177 | 0.808 | 0.602-1.083 | 0.154 |
| Quartile 4 | 0.83 | 0.628-1.096 | 0.189 | 0.988 | 0.74-1.32 | 0.937 |
| TSHI (continuous) | 0.973 | 0.846-1.117 | 0.694 | 1.056 | 0.915-1.218 | 0.457 |
| Quartile 1 | Ref |  |  |  |  |  |
| Quartile 2 | 0.961 | 0.728-1.268 | 0.776 | 0.947 | 0.71-1.263 | 0.711 |
| Quartile 3 | 1.044 | 0.797-1.367 | 0.755 | 0.929 | 0.702-1.23 | 0.606 |
| Quartile 4 | 0.914 | 0.695-1.203 | 0.522 | 0.946 | 0.711-1.259 | 0.705 |
| TT4RI (continuous) | 1.001 | 0.998-1.004 | 0.492 | 1.002 | 1-1.005 | 0.094 |
| Quartile 1 | Ref |  |  |  |  |  |
| Quartile 2 | 1.041 | 0.792-1.369 | 0.773 | 0.999 | 0.753-1.326 | 0.994 |
| Quartile 3 | 0.928 | 0.704-1.224 | 0.598 | 0.904 | 0.677-1.207 | 0.494 |
| Quartile 4 | 1.021 | 0.782-1.335 | 0.877 | 0.957 | 0.724-1.266 | 0.760 |

Abbreviation: HR, hazard ratio; CI, confidence interval; TFQI, thyroid feedback quantile-based index; PTFQI, Chinese-referenced parametric thyroid feedback quantile-based index; TSHI, thyrotropin index; TT4RI, thyrotroph thyroxine resistance index; BMI, body mass index; eGFR, estimated glomerular filtration rate.

Model 1: age and sex adjusted; model 2: age, sex, education level, BMI, physical activity, smoking, hypertension, hyperlipidemia, eGFR and fasting glucose adjusted.

**Table S6**: Cross-lagged standard regression coefficient of thyroid hormone sensitivity indices and fasting glucose after excluding 63 participants using anti-diabetic mediation.

|  | Model 1 | | | | Model 2 | | | |
| --- | --- | --- | --- | --- | --- | --- | --- | --- |
|  | β | P value | 95% CI | | β | P value | 95% CI | |
|  |  |  | lower | upper |  |  | lower | upper |
| **TFQI and Glucose** * |  |  |  |  |  |  |  |  |
| Glucose.x→TFQI.y | 0.040 | 0.000 | 0.023 | 0.061 | 0.028 | 0.014 | 0.008 | 0.050 |
| TFQI.x→Glucose.y | 0.011 | 0.107 | -0.006 | 0.023 | 0.010 | 0.298 | -0.014 | 0.029 |
| **PTFQI and Glucose** * |  |  |  |  |  |  |  |  |
| Glucose.x→PTFQI.y | 0.050 | 0.000 | 0.031 | 0.069 | 0.031 | 0.010 | 0.012 | 0.058 |
| PTFQI.x→Glucose.y | 0.016 | 0.076 | 0.001 | 0.036 | 0.012 | 0.135 | -0.007 | 0.025 |
| **TSHI and Glucose** * |  |  |  |  |  |  |  |  |
| Glucose.x→TSHI.y | 0.037 | 0.000 | 0.020 | 0.057 | 0.022 | 0.027 | 0.000 | 0.043 |
| TSHI.x→Glucose.y | 0.009 | 0.260 | -0.014 | 0.024 | 0.009 | 0.328 | -0.007 | 0.030 |
| **TT4RI and Glucose** |  |  |  |  |  |  |  |  |
| Glucose.x→TT4RI.y | 0.019 | 0.025 | 0.005 | 0.037 | 0.007 | 0.477 | -0.013 | 0.026 |
| TT4RI.x→Glucose.y | 0.006 | 0.530 | -0.017 | 0.021 | 0.007 | 0.384 | -0.011 | 0.022 |
| **FT3/FT4 and Glucose** * |  |  |  |  |  |  |  |  |
| Glucose.x→FT3/FT4.y | -0.033 | 0.001 | -0.052 | -0.015 | -0.024 | 0.022 | -0.044 | -0.001 |
| FT3/FT4.x→Glucose.y | -0.008 | 0.422 | -0.029 | 0.010 | -0.006 | 0.456 | -0.022 | 0.012 |

Abbreviation: CI, confidence interval; TFQI, thyroid feedback quantile-based index; PTFQI, Chinese-referenced parametric thyroid feedback quantile-based index; TSHI, thyrotropin index; TT4RI, thyrotroph thyroxine resistance index; FT3, free triiodothyronine; FT4, free thyroxine.

.x indicates measurements at baseline and .y indicates measurements at last time follow-up.

* indicates significant difference (P <0.001) in the cross-lagged coefficients using t test comparing the coefficients from thyroid sensitivity index to glucose and from glucose to thyroid sensitivity index.

Model 1: follow-up time adjusted; model 2: adjusted for age, sex, BMI and follow-up time.

**2 Supplemental Figures**


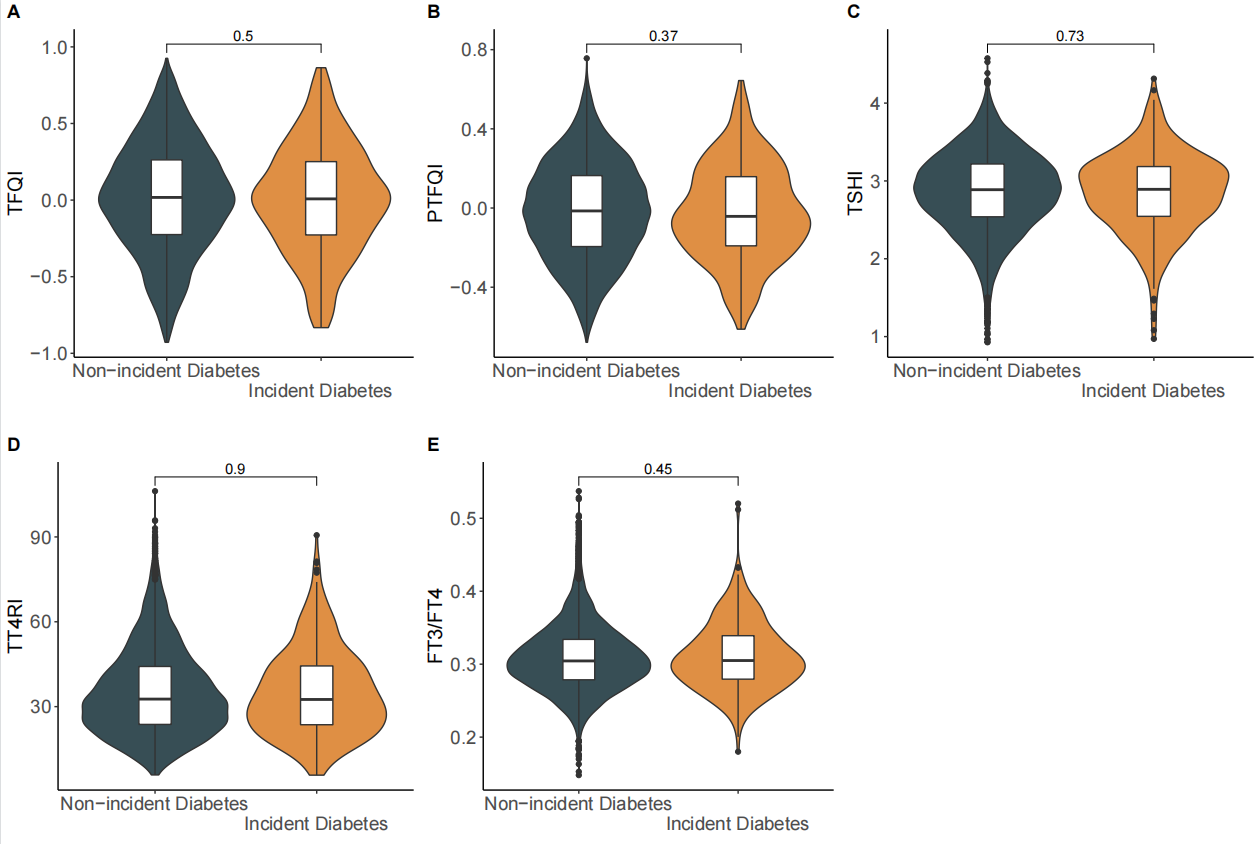


**Figure S1**: Distribution of baseline thyroid hormone sensitivity indices according incident diabetes or not.

P values were calculated using Mann-Whitney U test.


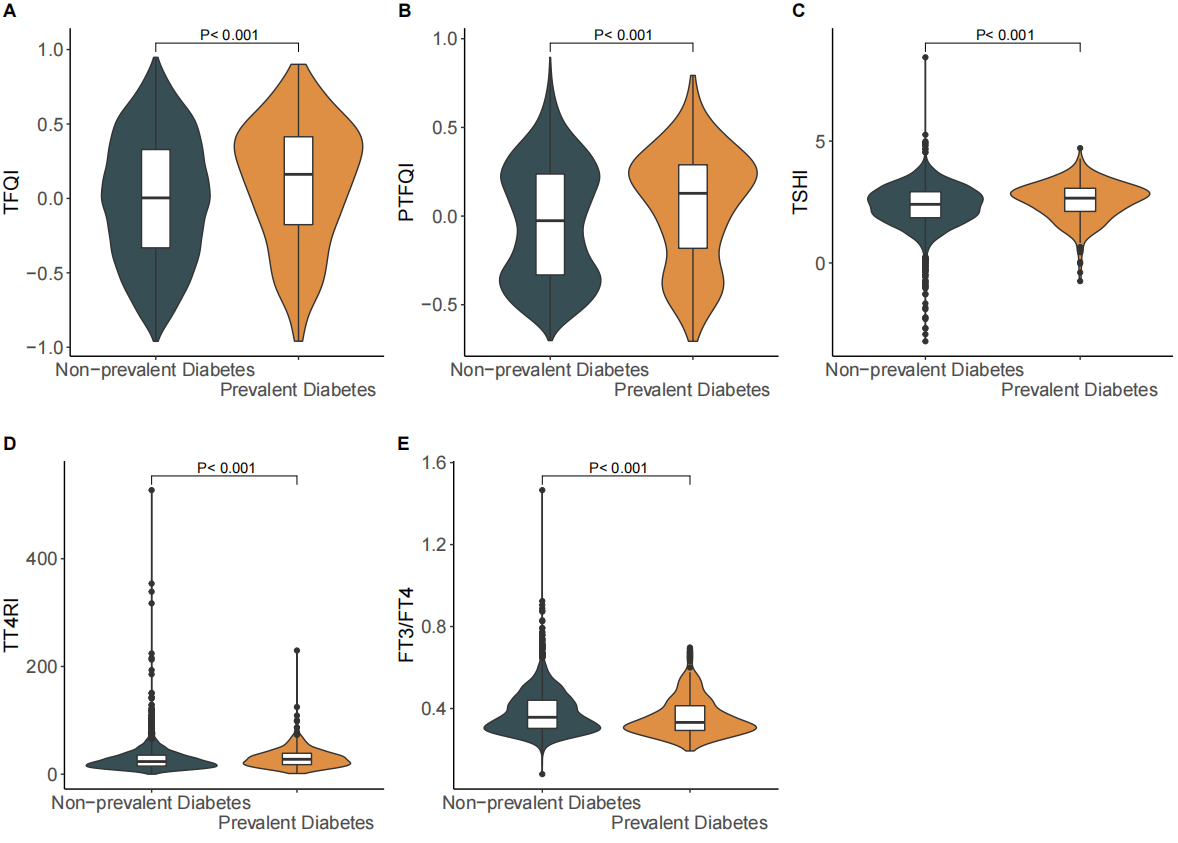


**Figure S2**: Distribution of thyroid hormone sensitivity indices according to prevalent diabetes or not at the last survey of follow-up.

P values were calculated using Mann-Whitney U test.


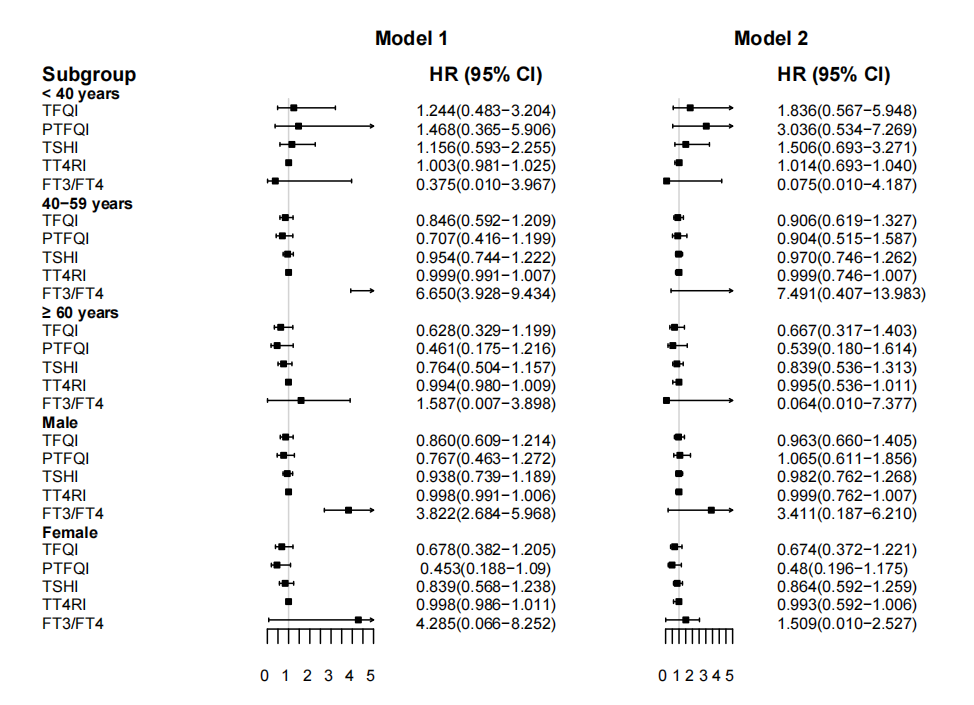


**Figure S3**: Association of baseline thyroid hormone sensitivity indices with incident diabetes among subgroups of age and sex.

Abbreviation: HR, hazard ratio (per unit); CI, confidence interval; TFQI, thyroid feedback quantile-based index; PTFQI, Chinese-referenced parametric thyroid feedback quantile-based index; TSHI, thyrotropin index; TT4RI, thyrotroph thyroxine resistance index; FT3, free triiodothyronine; FT4, free thyroxine; BMI, body mass index; eGFR, estimated glomerular filtration rate.

Model 1: age and sex (if not stratified) adjusted; model 2: age, sex (if not stratified), education level, BMI, physical activity, smoking, hypertension, hyperlipidemia, eGFR and fasting glucose adjusted.
